# Tamiflu-Resistant but HA-Mediated Cell-to-Cell Transmission through Apical Membranes of Cell-Associated Influenza Viruses

**DOI:** 10.1371/journal.pone.0028178

**Published:** 2011-11-30

**Authors:** Kotaro Mori, Takahiro Haruyama, Kyosuke Nagata

**Affiliations:** Department of Infection Biology, Faculty of Medicine and Graduate School of Comprehensive Human Sciences, University of Tsukuba, Tsukuba, Japan; Erasmus Medical Center, The Netherlands

## Abstract

The infection of viruses to a neighboring cell is considered to be beneficial in terms of evasion from host anti-virus defense systems. There are two pathways for viral infection to “*right next door*”: one is the virus transmission through cell-cell fusion by forming syncytium without production of progeny virions, and the other is mediated by virions without virus diffusion, generally designated cell-to-cell transmission. Influenza viruses are believed to be transmitted as *cell-free* virus from infected cells to uninfected cells. Here, we demonstrated that influenza virus can utilize cell-to-cell transmission pathway through apical membranes, by handover of virions on the surface of an infected cell to adjacent host cells. Live cell imaging techniques showed that a recombinant influenza virus, in which the *neuraminidase* gene was replaced with the *green fluorescence protein* gene, spreads from an infected cell to adjacent cells forming infected cell clusters. This type of virus spreading requires HA activation by protease treatment. The cell-to-cell transmission was also blocked by amantadine, which inhibits the acidification of endosomes required for uncoating of influenza virus particles in endosomes, indicating that functional hemagglutinin and endosome acidification by M2 ion channel were essential for the cell-to-cell influenza virus transmission. Furthermore, in the cell-to-cell transmission of influenza virus, progeny virions could remain associated with the surface of infected cell even after budding, for the progeny virions to be passed on to adjacent uninfected cells. The evidence that cell-to-cell transmission occurs in influenza virus lead to the caution that local infection proceeds even when treated with neuraminidase inhibitors.

## Introduction

It is generally accepted that viruses, released as *cell-free* virions from an infected cell, transmit to distant cells and tissues. This spreading pathway contributes to wide-ranged diffusion of *cell-free* viruses. However, in this spreading pathway, viruses are exposed to host anti-virus defense systems. In contrast, direct infection to a neighboring cell is considered to be beneficial for the virus in terms of evasion from the host anti-virus defense. There are two typical manners in infection to “*right next door*”: one is the virus transmission through cell-cell fusion by forming syncytium without production of progeny virions, and the other is mediated by virions without virus diffusion, generally designated cell-to-cell transmission [1], [2].

The cell-cell fusion infection pathway is characteristic for a variety of virus such as paramyxoviruses, herpesviruses, some retroviruses, and so on. For example in the case of measles virus belonging to *Paramyxoviridae*, infection is initiated by the interaction of the viral hemagglutinin glycoprotein with host cell surface receptors. The virus penetrates into the cell through membrane fusion mediated by the interaction of the fusion glycoprotein. In later stages of infection, newly synthesized glycoproteins accumulate at the cell membrane resulting in fusion of the infected cell with neighboring cells by producing syncytia. Thus, viruses can spread from cell to cell without producing *cell-free* virus particles.

The examples of the cell-to-cell transmission are diverse, and these mechanisms are dependent on pairs of viruses and host cells. Vaccinia virus particles bound on the filopodium of an infected cell are repelled toward neighboring uninfected cells by the formation of filopodia using actin filament [3]. The filopodia direct viruses to uninfected cells. Immunotropic viruses including retroviruses utilize an immunological synapse, designed as virological synapses for the cell-to-cell transmission [4]–[7]. Claudin-1 and occludin, components of tight junction, are involved in hepatitis C virus (HCV) entry through the cell-to-cell transmission [8], [9]. The cell-to-cell transmission through tight junction is also observed in other viruses which infect epithelial layers [10], [11]. These retroviruses and HCV remain on the surface of an infected cell even after budding. The uninfected cells adjacent to these infected cells can accept or take over viruses from the infected cell. Thus, the cell-to-cell transmission can be categorized into two manners based on the state of infecting viruses, either c*ell-free* or cell-associated virions.

Influenza virus, belonging to the family of *Orthomyxoviridae*, is one of the most serious zoonotic pathogens and causes seasonal epidemics or periodic pandemics among human beings around the world. The viral envelope consists of a lipid bilayer derived from cells that anchors three of viral transmembrane proteins, hemagglutinin (HA), neuraminidase (NA), and matrix protein 2 (M2). Influenza virus infection is initiated by the attachment of HA on virus particles to cell surface receptors containing sialic acids [12]. It has been known that the specific interaction between HA and sialic acid species is one of the determinants of the host range of influenza viruses [13]. Beside its role in the viral attachment, HA is also involved in intracellular fusion between viral envelope and host cell endosome membrane in the endocytotic pathway, by which the virus content is released inside the host cell [14]. The functional maturation of HA is mediated by the cleavage of HA into two disulfide-linked glycopolypeptides, HA1 and HA2 [15], accomplished by trypsin or trypsin-like proteases derived from host cells [16]–[19]. The membrane fusion is induced by a conformational change in the mature HA, which is triggered at low pH in the endosome, allowing viral ribonucleoprotein complexes to release into the cytoplasm [20], [21]. Thus, HA plays a critical role in initiation and progression of influenza virus infection. Influenza virus NA possesses the enzymatic activity that cleaves α-ketosidic linkages between terminal sialic acids and adjacent sugar residues of cellular glycoconjugates [22]. The sialidase activity of NA removes terminal sialic acid residues from HA and NA proteins as well as host cell surface glycoproteins. Since the terminal sialic acid of sialyloligosaccharides is critical for HA binding, the receptor-destroying activity of NA serves to counter the receptor-binding activity of HA. It is quite likely that this activity contributes to prevention of successive superinfection of an infected cell [23]. In the absence of the functional sialidase activity, progeny virions aggregate on the cell surface due to the HA receptor-binding activity and can not be released [24], [25]. Thus, NA cleaves sialic acids from the cell surface and facilitates virus release from infected cells. However, it is not clear whether every progeny virion is released as *cell-free* virion to infect the uninfected cells after diffusion into the extracellular environment. Influenza viruses are generally transmitted as *cell-free* viruses from infected to uninfected cell but they may also infect through the cell-to-cell transmission, in particular during local lesion formation.

Here, we examined whether influenza virus transmits from an infected cell to adjacent uninfected cells without virus release. Live cell imaging techniques showed that a recombinant influenza virus, in which the *NA* gene was replaced with the *green fluorescence protein* gene, spreads from an infected cell to adjacent cells forming infected cell clusters. Furthermore, progeny virions remain associated on the surface of infected cell even after budding, and then progeny virions could be passed to adjacent uninfected cells.

## Results

### Influenza virus can spread in an NA-independent manner to adjacent cells

**Figure 1:**
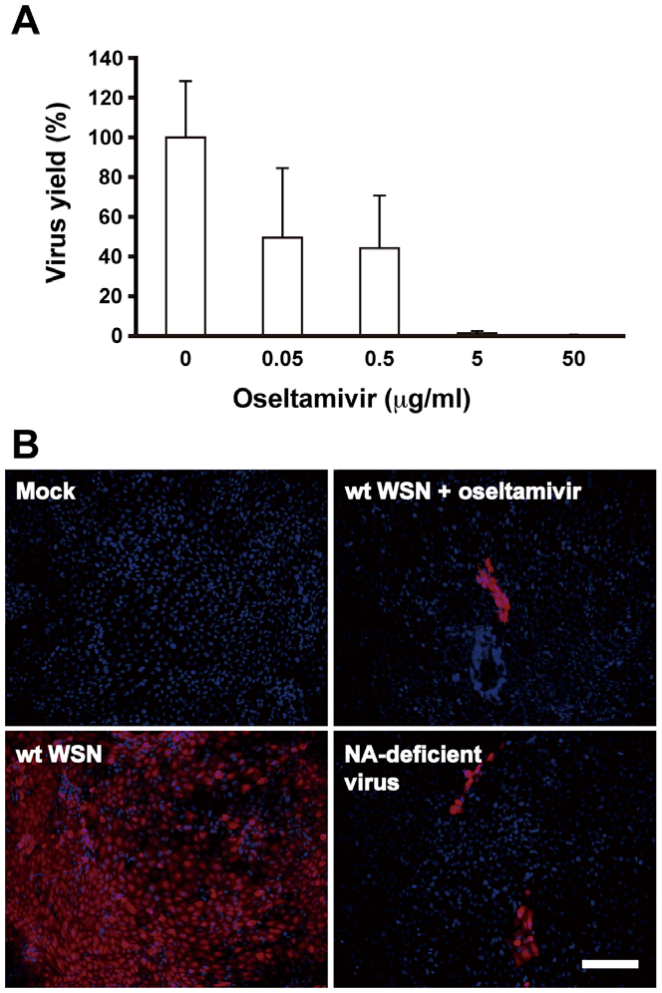
Influenza viruses can spread independent of the NA activity. (A) MDCK cells were infected with influenza virus A/WSN/33 at a multiplicity of infection (MOI) of 0.001 PFU per cell. At 48 hours post infection (hpi), culture supernatant was collected, and then its virus titer was determined by plaque assays. Each result was represented by a value relative to that in the absence of the drug. Error bars indicate standard deviation (s.d.) from 3 independent experiments. (B) Confluent MDCK cells were infected by wild-type influenza virus A/WSN/33 or NA-deficient influenza virus at MOI of 0.0001 in the presence or absence of 50 µg/ml oseltamivir phosphate. NA-deficient influenza virus was generated by reverse genetics as previously described [29]. After incubation at 37°C for 36 hours, immunofluorescence analyses were performed using anti-nucleoprotein (NP) polyclonal antibody and anti-rabbit IgG antibody conjugated to Alexa Fluor 568 (Invitrogen). Scale bar, 100 µm.

To examine the transmission pathway of influenza virus, we performed immunofluorescence analyses by using anti-nucleoprotein (NP) polyclonal antibody. Influenza virus can form an infection center even in the presence of oseltamivir, a potent NA inhibitor (commercially known as Tamiflu) [26]–[28]. Oseltamivir at the concentration of 50 µg/ml completely prevented the release of progeny influenza viruses (Figure 1A). Noted that a large number of single fluorescent foci caused by initial infection markedly expanded and formed cell clusters consisting of 5–10 infected cells in an MDCK cell monolayer (Figures 1B and S1), suggesting influenza virus can spread to some extent in the presence of oseltamivir. To verify that NA is not involved in this spreading, we generated an NA-deficient influenza virus by a reverse genetics method as described previously [29], [30]. The NA-deficient influenza virus contains a mutated NA segment, in which the NA coding region including a sialidase catalytic domain was replaced with the *enhanced green fluorescent protein (EGFP)* gene [29]. By this replacement, the NA activity is eliminated from the recombinant influenza virus, and *EGFP* can be utilized as a marker for viral infections. Immunofluorescence analyses demonstrated that the NA-deficient influenza virus also forms infected cell clusters similarly to those formed by wild-type influenza virus in the presence of oseltamivir (Figure 1B). The fluorescence pattern of NP overlapped with the localization of GFP derived from the *EGFP* gene of the NA-deficient influenza virus (Figure S2). Thus, NA-deficient influenza virus can be used to investigate the NA-independent infection pathway of influenza virus.

**Figure 2:**
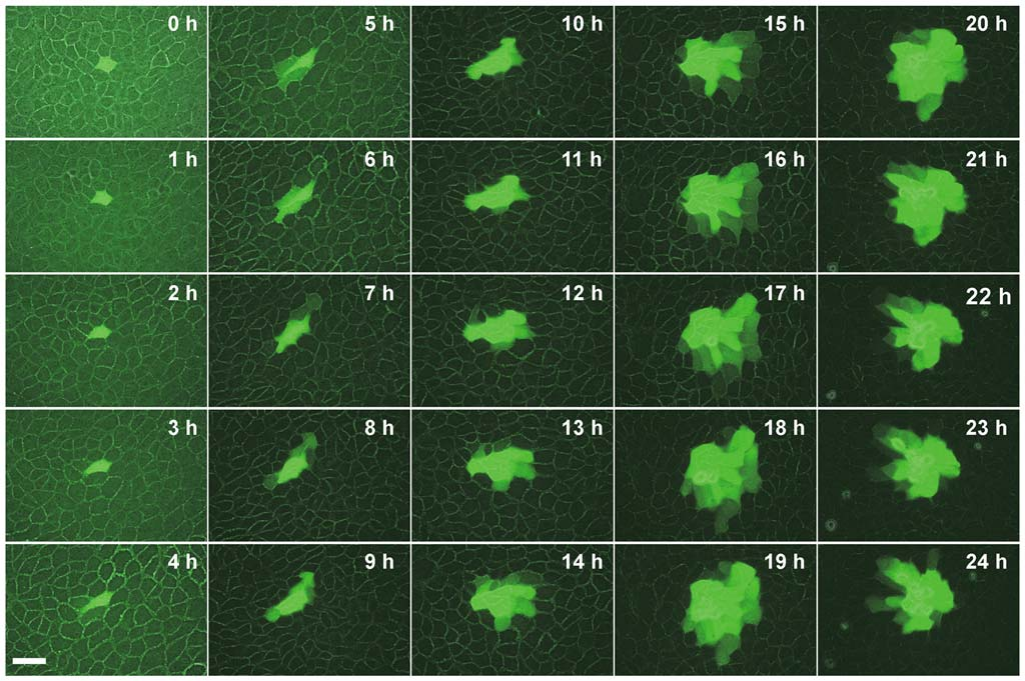
NA-deficient influenza virus spreads through cell-to-cell transmission. Confluent MDCK cells were infected with the NA-deficient influenza virus at MOI of 0.0001. After incubation at 37°C for 24 hours, a single GFP-positive cell, in which the recombinant virus replicated, was found at 1 hour after starting monitoring, and then this cell and its neighborhood were traced during the period from 24 hpi to 48 hpi at interval of 1 hour. Scale bar, 50 µm.

Next, we performed live cell imaging analyses to directly observe the infection time course of the NA-deficient influenza virus. The GFP fluorescence derived from the NA-deficient influenza virus first appeared in a single cell on an MDCK cell monolayer at 24 hours post infection. The virus started to spread from an infected cell to adjacent cells in 5–6 hours after the first appearance of a GFP-positive cell (Figure 2 and Video S1). The spreading rate was clearly faster than the rate of cell divisions. The mean doubling time of uninfected MDCK cells was 20–24 hours under the condition employed here, and it is expected that the proliferation speed would be much slowly because infected MDCK cells were maintained in the serum-free medium and formed cell monolayer at the high cell density. These suggest that NA-deficient influenza viruses may infect adjacent cells through the cell-to-cell transmission mechanism without apparent production of *cell-free* virions.

### Cell-to-cell transmission pathway of influenza viruses is less sensitive to neutralizing antibody

**Figure 3:**
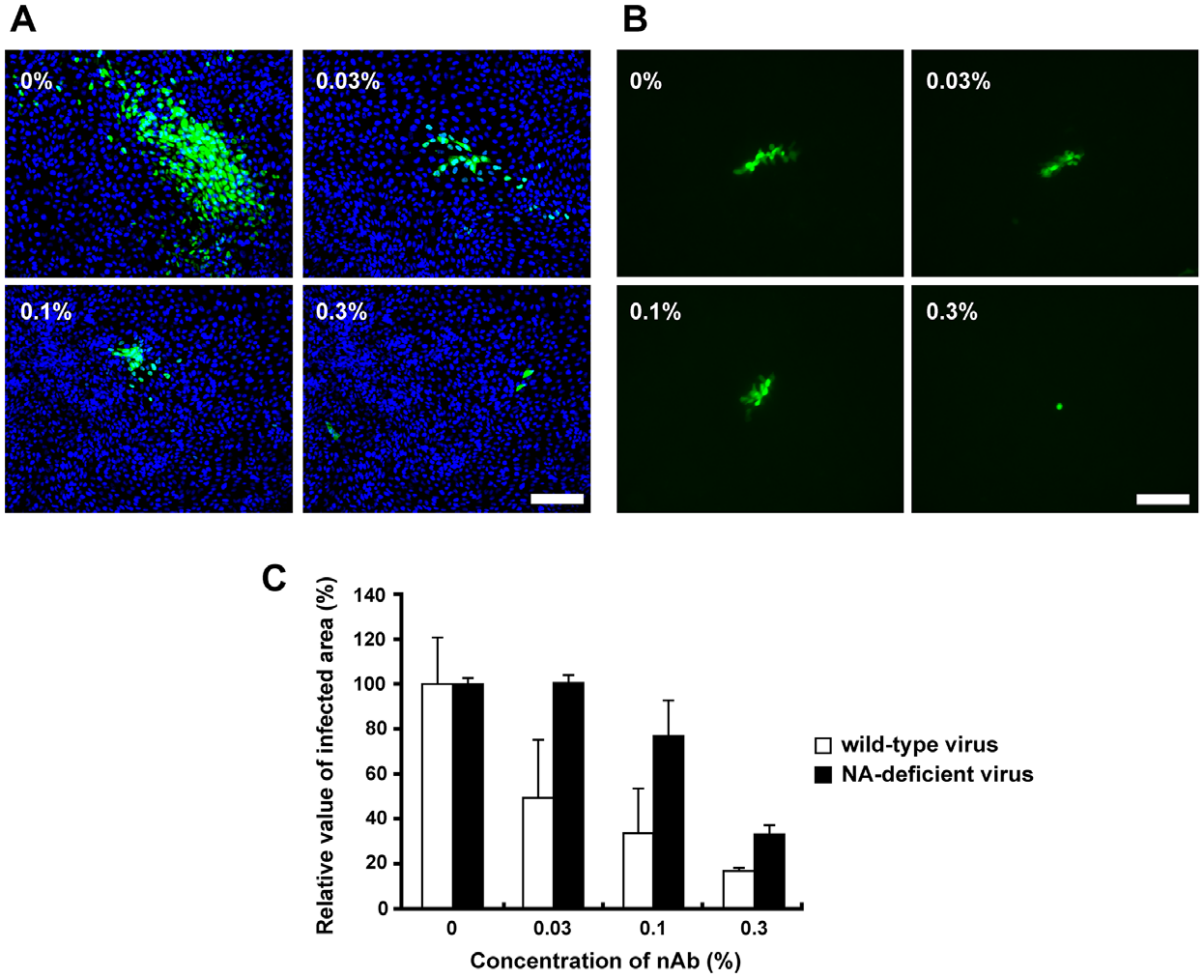
The cell-to-cell transmission of the NA-deficient influenza virus is less sensitive to the neutralizing antibody. (A) Infection of the wild-type and (B) NA-deficient influenza virus were performed in the presence or absence of antiserum containing neutralizing antibodies. Immunofluorescence analyses were performed with cells infected with wild-type influenza virus at 18 hpi using anti-NP antibody and anti-rabbit IgG antibody conjugated to Alexa Fluor 488 (Invitrogen). GFP fluorescence derived from the recombinant virus was observed at 36 hpi. Scale bar, 100 µm. (C) The level of viral spreading was indicated in the graph by measuring NP and GFP derived from wild-type and NA-deficient virus, respectively. Five different microscope fields were taken randomly, and then the intensity of green color was analyzed with ImageJ NIH image processing software. Each result was represented by a value relative to that in the absence of neutralizing antibodies. Error bars indicate s.d. from 3 independent experiments.

The cell-to-cell virus transmission pathway could be interpreted as one of viral evolving strategies to avoid neutralizing antibody responses [2], [31], [32]. Therefore, we examined the effect of neutralizing antibody on NA-deficient influenza virus. A polyclonal antibody with the neutralizing activity against influenza virus particles inhibited infection of *cell-free* viruses to less than 50% at the concentration of 0.03%, although the cell cluster formation was observed at the concentration less than 0.01%. On the other hand, the NA-independent transmission of the NA-deficient influenza virus was blocked only when neutralizing antibody was present at the concentration of 0.3% (Figure 3). These results indicated that the NA-independent transmission of influenza viruses is less sensitive to the neutralizing antibody.

### NA-independent transmission of influenza virus is HA-dependent

**Figure 4:**
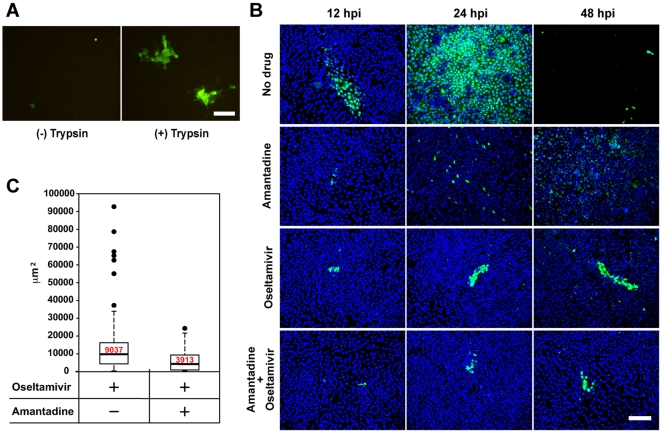
The cell-to-cell transmission of the NA-deficient influenza virus requires functional HA. (A) Confluent MDCK cells were infected with the NA-deficient influenza virus at MOI of 0.0001 in the presence or absence of 1 µg/ml trypsin. GFP fluorescence derived from the recombinant virus was observed at 36 hpi. Scale bar, 100 µm. (B) MDCK cells were infected with influenza virus A/Udorn/72 at moi of 0.0001 in the presence or absence of 50 µM amantadine or 50 µg/ml oseltamivir phosphate. Amantadine at the concentration of 50 µM almost completely inhibited the production of progeny virions (data not shown). After incubation for 12, 24, and 48 h, immunofluorescence analyses were performed using anti-NP antibody and anti-rabbit IgG antibody conjugated to Alexa Fluor 488 (Invitrogen). Viral NP and nuclear DAPI staining are shown in green and blue, respectively. Scale bar, 100 µm. (C) Median sizes of clusters were shown as box plots summarizing sizes of 60 individual infectious foci formed in the presence of oseltamivir alone, or both oseltamivir and amantadine. Immunofluorescence analyses were performed as described in (B) at 24 hpi. Boxes enclose the lower and upper quartiles; thick horizontal lines represent the median; dashed lines indicate the extreme values; and black dots are outliers of individual infectious foci. The size of infectious foci was measured with AxioVision Release 4.7.2 imaging software (Carl Zeiss). Median sizes shown in red letters were clearly different from each other (p<0.01).

Next, to investigate the mechanism of NA-independent transmission of influenza virus, we examined whether HA is involved in this transmission. In the absence of the NA activity, virus spreading from an infected cell to adjacent cells was dramatically suppressed by omission of trypsin, essential for maturation of HA, from the experimental condition (Figure 4A). The GFP fluorescence derived from NA-deficient influenza virus appeared in a single cell at 24 hours post infection. However, this virus did not spread, but rather disappeared during subsequent 24 hours (Video S2). These observations indicate that the NA-independent cell-to-cell transmission of influenza virus is dependent on HA maturation mediated by trypsin, as is the case for the general *cell-free* transmission of this virus.

To clarify whether virus particles or viral RNP complexes are transmitted to adjacent cells, we examined the effect of amantadine on the cell-to-cell transmission of influenza virus. Amantadine inhibits the early step of uncoating of influenza virus RNP from virion in endosomes [33], [34]. For this study, other influenza virus strain, influenza virus A/Udorn/72, was used instead of influenza virus A/WSN/33 because influenza virus A/WSN/33 is highly resistant to amantadine [35]. We confirmed that influenza virus A/Udorn/72 is sensitive to oseltamivir (Figure S3) and could also spread via cell-to-cell transmission independent of the NA activity as did for influenza virus A/WSN/33 (Figures 1B and 4B). In the case of a single administration of amantadine, fluorescent foci derived from infected cells scattered, and the number of single foci was greatly decreased compared with that in the absence of the drugs. In contrast, a single administration of oseltamivir, fluorescent foci formed some clusters and expanded in a time-dependent manner (Figure 4B). This dissimilarity of inhibitory manner was caused by the difference of the sites of action between amantadine and oseltamivir. Amantadine inhibits the replication of influenza A virus by preventing the translocation of vRNP complexes from endosomes to the cytoplasm, whereas oseltamivir has no effects on viral replication itself but inhibits the release of *cell-free* virions from infected host cells. We investigated the inhibitory effect of amantadine on the cell-to-cell transmission of influenza viruses. The formation of infected cell clusters was observed with co-administration of amantadine and oseltamivir, as well as with a single administration of oseltamivir (Figure 4B). However, the quantitative analysis revealed that the size of infected cell clusters with the co-administration were decreased as compared to that with oseltamivir alone (Figure 4C). These observations indicated that the NA activity-independent cell-to-cell transmission of influenza virus was susceptible to the inhibitory effect of amantadine, suggesting that the cell-to-cell transmission undergoes through endocytosis but vRNP complex itself is not incorporated in the infected cells by adjacent cells.

### Cell-to-cell transmission occurs on the apical cell membrane

The virus transmission undergoes from infected to uninfected cells through either basolateral [36]–[38] or apical [39]–[42] sides. In the case of influenza virus, *cell-free* progeny virions are released only from the apical surface of polarized epithelial cells [43]. This releasing polarity is achieved by directed transport of viral membrane proteins to the apical plasma membrane [44]. Indeed, that HA and NA glycoproteins are associated with lipid rafts, and the raft association has been implicated in apical transport [45], [46].

**Figure 5:**
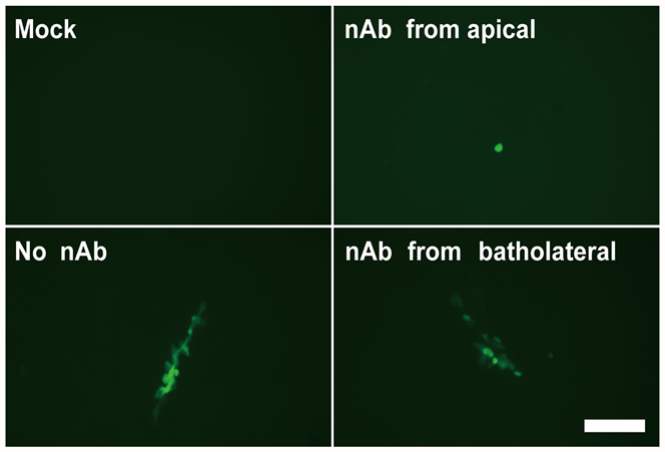
The cell-to-cell transmission of the NA-deficient influenza virus occurs the apical cell surface. Confluent MDCK cells were prepared in transwell inserts and infected with the NA-deficient influenza virus at MOI of 0.0001 in the presence or absence of 0.3% (v/v) antiserum containing neutralizing antibodies (nAb) to influenza A virus. After virus adsorption, the antiserum was added from apical or basolateral side. GFP fluorescence derived from the recombinant virus was observed at 36 hpi. The antiserum added from the apical side could markedly block the cell-to-cell transmission of the NA-deficient influenza virus, whereas the antiserum added from the basolateral side could not. Scale bar, 100 µm.

To determine whether or not the cell-to-cell transmission of the NA-deficient influenza virus occurs on the apical surface, we performed transwell assays in the presence of the neutralizing antibody to influenza A viruses. The neutralizing antibody was added to infected MDCK cell monolayer from apical or basolateral side, and the inhibitory effect on the spread of GFP fluorescence derived from the recombinant virus was examined. Addition of high concentrations of the neutralizing antibody from the apical side blocked the cell-to-cell transmission of the NA-deficient influenza virus, whereas the addition from the basolateral side had no effect (Figure 5). These observations indicated that the polarity in the influenza virus budding in the cell-to-cell transmission pathway is apical.

### Influenza viruses can not re-infect previously infected cells

**Figure 6:**
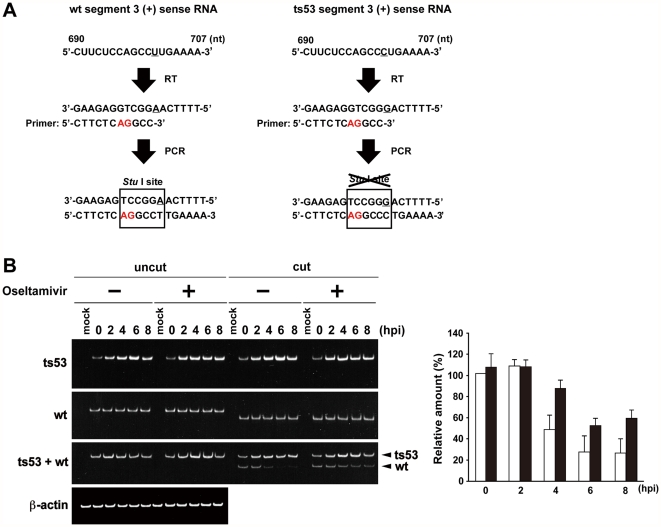
Influenza viruses can not re-infect previously infected cells. (A) A method for determination of the amount of segment 3 genome derived from *ts*53 and wild-type. Total RNA was reverse-transcribed with the primer PA-895-rev, which is complementary to the segment 3 positive-sense RNA. The cDNA was amplified by PCR using primers, PA-895-rev and PA-695-cut partially corresponding to segment 3 positive sense RNA between the nucleotide sequence positions 678 to 700 except for 696 and 697, which are shown in red letters. Since segment 3 of *ts*53 has a substitution mutation from U to C at the nucleotide position of 701, the PCR product derived from wild-type could be digested by *Stu* I but not that from *ts*53. Then, PCR products were digested with *Stu* I and separated through 8% PAGE. (B) Detection of the genome of the segment 3 derived from *ts*53 or wild-type. At 3 hours post superinfection of wild-type virus, total RNA was extracted, and semi-quantitative RT-PCR was performed. Subsequently, the amplified DNA products were digested with *Stu* I and separated through 8% PAGE. Large and small fragments derived from *ts*53 and wild-type viruses were 220 and 199 base pairs, respectively. The relative amount of wild-type segment 3 to that at 0 hour in the absence of oseltamivir phosphate was shown in the graph. Error bars indicate S.D. from 3 independent experiments. White bar, in the absence of oseltamivir phosphate; black bar, in the presence of oseltamivir phosphate.

Previous report showed that influenza viruses were refractory to superinfection with a second cell-free virus [23]. In the case of the cell-to-cell transmission of influenza virus in the presence of oseltamivir, it is possible that a progeny virion is temporarily bridged by HA between an infected cell and adjacent uninfected cells, since viruses can not be released from infected cell surface due to the inhibition of the NA activity by oseltamivir. The cell-associated progeny virion may have an opportunity to re-infect the previously infected cell, compared to a cell-free progeny virion in the general spreading. Thus, we examined whether influenza viruses can infect the cell which had already been infected, using *ts*53 mutant and wild-type influenza virus A/WSN/33. *ts*53 virus has a substitution mutation from U to C at the nucleotide position of 701 in the PA gene. This substitution introduces an amino acid change from wild-type Leu 226 to Pro 226 and gives a defect in the viral genome replication process [47], [48]. At first, cells were infected with *ts*53 virus at moi of 10, and after incubation for 0, 2, 4, 6, and 8 hours, cells were superinfected with wild-type virus at moi of 10. The amount of segment 3 viral RNA (vRNA) encoding PA was determined quantitatively by RT-PCR. Then, using a mutated primer for PCR, we could introduce a *Stu* I site only in the PCR products derived from the wild-type sequence (Figure 6A). Thus, DNA fragments amplified from the wild-type and *ts*53 could be distinguished by *Stu* I digestion. The digested DNA fragments containing 220 and 199 base pairs derived from *ts*53 and wild-type, respectively, were separated through PAGE. After 6 hours or later post infection, re-infection with the second challenging virus hardly occurs in the absence of oseltamivir. However, in the presence of oseltamivir, appearance of wild-type fragment suggests that the re-infection had occurred (Figure 6B). The result indicates that progeny virus particles remain on the surface of infected cell even after budding, and can infect the cell previously infected, as well as uninfected cells adjacent to the infected cell, when oseltamivir is present.

## Discussion

With the except for the virus which spreads through the cell-cell fusion transmission, virus infection is initiated by the binding of *cell-free* virions to their host cells. Recently, the virus transmission mechanism from an infected cell to adjacent cells without virus diffusion into the extracellular environment is highlighted from the aspect of its significance in virus spreading in the presence of antibodies [1], [2]. This antibody-insensitive pathway is often called cell-to-cell transmission [2]. The cell-to-cell transmission may be categorized into two pathways, *i.e.*, transmission of *cell-free* virions to adjacent uninfected cells, and transmission of progeny virions associated on the surface of an infected cell even after budding through narrow synaptic space between an infected cell and adjacent uninfected cells. As an example of the former mechanism, *cell-free* vaccinia virus particles associated with the filopodium of an infected cell are repelled toward neighboring uninfected cells by inducing the formation of actin filament [3]. Several cases have been reported for the latter mechanism: Immunotropic viruses including retroviruses utilize the immunological synapses [4]–[7]. Immune cells are not constitutively polarized, but contain the machinery that directs their secretory apparatus towards a cell that is involved in an immunological synapse. This machinery can be subverted by retroviruses containing human immunodeficiency virus (HIV). An HIV-infected cell can polarize viral budding towards a target cell expressing receptor through a structure called a virological synapse. Virions bud from an infected cell into a synaptic cleft, from which they fuse with the target-cell plasma membrane [49]–[52]. The progeny virions of HCV are trapped between infected and uninfected cell membranes at the tight junction. Using Claudin-1 known as a component of the tight junction and one of the entry factors of HCV [8], virions fuse with and penetrate uninfected target cells [31]. Therefore, HCV may acquire the ability to spread within polarized liver epithelium. Thus, the cell-to-cell transmission certainly plays significant roles for the dissemination of several enveloped viruses. However, the cell-to-cell transmission of influenza virus has not been discussed well. Here, we have shown that influenza virus spreads by forming infected cell clusters even in the presence of an NA inhibitor. Live cell imaging clearly showed that influenza virus lacking the NA activity spreads from an infected cell to adjacent cells through the cell-to-cell transmission mechanism (Figure 2). This was also the case for wild-type influenza virus during early phases of infection (Figure 4B). In the cell-to-cell transmission of influenza virus, progeny virions could remain associated with the surface of infected cell even after budding, and then these progeny virions can be passed on to adjacent uninfected cells.

We showed that the cell-to-cell transmission of the NA-deficient influenza virus depends on functional HA. The viral spreading was dramatically suppressed without HA activation by trypsin treatment (Figure 4A). Moreover, the cell-to-cell transmission was also blocked by amantadine, which inhibits the acidification of endosomes required for uncoating of influenza virus particles in endosomes [33], [34]. These findings indicate that functional HA and endosome acidification by M2 ion channel are required for the cell-to-cell influenza virus transmission, thereby allowing viruses to enter the adjacent cells through the endocytotic pathway (Figure 4).

Our findings showed that the NA-deficient influenza virus is not diffused into the extracellular environment. The viral spreading in the absence of oseltamivir appears to be much faster compared to the viral spreading in the presence of the drug, suggesting that NA could be involved in determination of spreading speed (Figure 4B). The NA activity prevented progeny virions from entering cells which virus came from (Figure 6), implying that progeny virus particles should be transmitted to adjacent uninfected cells. The cell-to-cell transmission started in early phase of infection, and the virus spread through diffusion of *cell-free* viruses (Figure 4B). Indeed, it was reported that the cell-to-cell transmission is a rapid spreading pathway in the case of vaccinia virus [3]. Vaccinia virus induces a blocking mechanism of superinfection and thereby infects to adjacent uninfected cells efficiently. In early phases of vaccinia virus infection, viral proteins A33 and A36 are expressed at the infected cell surface. Once *cell-free* virus particles contact the filopodium, the A33/A36 complex induces the formation of actin filament, which causes this superinfected virion to be repelled toward uninfected cells [3]. Influenza viruses can re-infect the cells previously infected in the presence of oseltamivir (Figure 6), suggesting that a progeny virion may be bridged by HA between infected and adjacent uninfected cells temporarily. Thus, in the case of the cell-to-cell transmission of influenza virus, we propose that progeny virions associated with the surface of infected cells even after budding are directed to adjacent uninfected cells. The cell-to-cell transmission mechanism of influenza virus is distinctly different from that of vaccinia virus in the infecting virus status: Infected cell-associated virions and *cell-free* virions are involved in the cell-to-cell transmission of influenza virus and vaccinia virus, respectively. The strategy for influenza virus appears to be similar to that for HCV. HCV progeny virions budded from an infected cell are trapped between infected and uninfected adjacent cell membranes at the tight junction. HCV virions then, enter into adjacent cells through endocytosis and low pH-dependent membrane fusion using Claudin-1 [8]. The cell-to-cell transmission of influenza virus also required functional HA and endosome acidification by M2 ion channel. However, it has not been reported that HCV has a gene encoding a receptor destroying enzyme similar to NA of influenza virus. We speculated that HCV progeny particles are bridged between infected and adjacent uninfected cells temporarily like influenza virus in the presence of oseltamivir. Progeny influenza virus particles could be transmitted to adjacent uninfected cells efficiently in the presence of the NA activity, suggesting that the cell-to-cell transmission of influenza virus is more strategic than that of HCV.

Our findings raise an interesting question as to what is the biological significance of cell-to-cell transmission for influenza virus infection *in vivo*. Until now, it had been believed that influenza virus was released from infected cells as *cell-free* virions and then spread from cell to cell as well as from organism to organism. The transmission mode by *cell-free* virions undergoes the extremely high-speed of its diffusion and causes epidemic or pandemic infection. The tropism in an infected animal body is generally restricted to respiratory tract or lung and its periphery, and the requirement of a trypsin-like protease has been generally described for the reason of the restriction. It is possible that the cell-to-cell transmission mode may play a significant role for the virus spreading inside of organism, although *cell-free* influenza virions are causative of high-speed spreading. At the least, the limited but distinct level of infection followed by replication could provide some opportunity to generate influenza virus variants. It is an open question whether the cell-to-cell transmission mode is involved in the pathogenesis caused by influenza virus infection *in vivo*.

The existence of cell-to-cell transmission pathway gives a caution when NA inhibitors are used, because NA inhibitors may not be sufficient to completely block the spread of influenza virus in local microenvironments. Since this cell-to-cell transmission pathway exists, development of antiviral therapeutic strategies in addition to NA inhibitors is highly recommended.

## Materials and Methods

### Cells and viruses

Madin-Darby canine kidney (MDCK) cells were kindly gifted by A. Ishihama (Hosei University), and maintained in minimal essential medium (MEM) (Nissui) containing 10% fetal bovine serum. Human embryonic kidney 293T cells were kindly gifted by Y. Kawaoka (University of Tokyo), and maintained in Dulbecco modified Eagle medium (DMEM) (Nissui) supplemented with 10% fetal bovine serum. Influenza virus A/Udorn/72 was grown in allantoic sacs of 11 day-old embryonated eggs (MIYAKE HATCHERY). Wild-type influenza virus A/WSN/33 and *ts*53 mutant were used after single-plaque isolation. MDCK cells were infected with influenza virus A/WSN/33 or *ts*53 at a multiplicity of infection (MOI) of 0.1 PFU/cell, and incubated at 37°C and 34°C, respectively. After incubation for 24 h, the culture fluid was harvested and centrifuged at 1,700× *g* for 10 min. The virus suspension was stored at −80°C until use.

### Antibodies

The production of rabbit polyclonal anti-NP antibody was described previously [53], and this antibody was used as a primary antibody for indirect immunofluorescence assay. A goat anti-rabbit IgG antibody conjugated to Alexa Fluor 488 or Alexa Fluor 568 was purchased from Invitrogen and used as a secondary antibody for indirect immunofluorescence assay. A polyclonal antibody against influenza A virus was obtained from 2-month-old female rabbit immunized with 250 µg of purified virions of influenza virus strain A/Puerto Rico/8/34 [54]. The generation of antibodies was boosted three times and used as neutralizing antibodies to block the influenza virus infection.

### Determination of the inhibition effect of oseltamivir on virus production

MDCK cells were infected with influenza virus A/WSN/33 at a multiplicity of infection (MOI) of 0.001 PFU per cell. After virus adsorption at 37°C for 1 hour, the cells were washed with serum-free MEM and incubated at 37°C with maintenance medium (MEM containing vitamins and 0.1% BSA) containing oseltamivir. At 48 hours post infection (hpi), culture supernatant was collected, and then its viral titer was determined by plaque assays.

### Generation of neuraminidase (NA)-deficient viruses

An NA-deficient influenza virus possessing the terminal sequences of NA segment but lacking the NA coding region, which was replaced with *enhanced green fluorescent protein (EGFP)* gene, was generated by reverse genetics as described previously [29], [30]. For reverse genetics, we used plasmids containing cDNAs of the influenza virus A/WSN/33 viral genome under the control of the human RNA polymerase I promoter (referred to as Pol I plasmids). Briefly, 293T cells were transfected with seven Pol I plasmids for production of all vRNA segments of influenza virus A/WSN/33 and one for the mutant NA vRNA segment containing *EGFP* ORF, together with protein expression vectors for PB2, PB1, PA, and NP controlled by the chicken β-actin promoter (pCAGGS). TransIT-293 (Mirus) was used for transfection. At 24 hours post transfection, recombinant viruses were harvested from the cell surface using bacterial NA derived from *Clostridium perfringens* (sigma). MDCK cells were infected with harvested recombinant viruses treated with *N*-tosyl-L-phenyl-alanine chloromethyl ketone (TPCK)-trypsin (1 µg/ml). After confirmation of GFP fluorescence derived from amplified recombinant virus genomes at 48 hours after infection, the recombinant viruses on the cell surface were collected using bacterial NA. The viral titer of recombinant viruses was determined by counting the number of infected foci using a fluorescence microscopy (Carl Zeiss).

### Indirect immunofluorescence assay

Cells on coverslips were fixed with 4% paraformaldehyde in phosphate-buffered saline (PBS) for 10 min and permeabilized with 0.2% NP-40 in PBS. The coverslips were soaked in 1% bovine serum albumin in PBS, and then incubated at room temperature for 1 hour with a primary antibody. After being washed twice with PBS, the coverslips were incubated at room temperature for 1 hour with a secondary antibody. The coverslips were then incubated at room temperature for 5 min with 3 µM 4′,6′-diamidino-2-phenylindole (DAPI) and finally mounted on glass plates, and cells were observed under the fluorescence microscope.

### Live cell imaging analyses

Living cells were analyzed using BioStation ID system (GE Healthcare). Confluent MDCK cells were infected with the NA-deficient influenza virus at the multiplicity of infection (MOI) of 0.0001 in the presence or absence of 1 µg/ml TPCK-trypsin. At 24 hours post infection, culture dishes containing infected cells were set into the chamber of BioStaion ID system, which was maintained at 37°C under 5% CO_2_ and 95% humidity. Then, images were acquired during next 24 hours at interval with 1 hour. The excitation wavelength was controlled by a manual filter wheel equipped with filters suitable for enhanced green fluorescence protein (EGFP).

### Transwell assay

Confluent MDCK cell monolayer was prepared on transwell inserts (BD Falcon, pore size 0.4 µm) and infected with the NA-deficient influenza virus at MOI of 0.0001. After virus adsorption at 37°C for 1 hour, the cell monolayer was washed with serum-free MEM, and maintenance medium was added into both sides within the transwells. The neutralizing antibody to influenza A virus was added into the inside or the outside of transwell inserts with the maintenance medium. Subsequently, cells were incubated at 37°C for 36 hours followed by analyses using the fluorescence microscopy.

### RT-PCR

*ts*53 virus has a substitution mutation from U to C at the nucleotide position of 701 in the *PA* gene. This substitution introduces an amino acid change from wild-type Leu 226 to Pro 226 and gives a defect in the viral genome replication process [48]. However, under the permissive temperature, the level of viral genome replication is no difference between wild-type and *ts*53 [47]. To discriminate the genome of wild-type and that of *ts*53, total RNA was reverse-transcribed by reverse transcriptase (TOYOBO) with PA-895-rev (5′-TTAATTTTAAGGCATCCATCAGCAGG-3′), which is complementary to the segment 3 positive sense RNA. The cDNA was amplified by PCR using primers, PA-895-rev and PA-695-cut (5′-TCTCCCGCCAAACTTCTCAGGCC-3′) partially corresponding to segment 3 positive sense RNA between nucleotide sequence positions 678 to 700 except for nucleotide positions 696 and 697. Since segment 3 of *ts*53 has a substitution mutation from U to C at the nucleotide position of 701, the PCR product derived from wild-type was digested by *Stu* I but not that from *ts*53. After PCR reactions, PCR products were digested with *Stu* I and separated through PAGE. Large and small fragments derived from *ts*53 and wild-type viruses were 220 and 199 base pairs, respectively. DNA was stained with GelRed (BIOTIUM) and visualized by UV illumination.

## Supporting Information

Figure S1 **Formation of cell cluster caused by initial infection.** MDCK cells were infected with influenza virus A/WSN/33 at moi of 0.0003 in the presence or absence of 50 µg/ml oseltamivir phosphate. After incubation for 8 and 24 h, immunofluorescence analyses were performed using anti-NP antibody and anti-rabbit IgG antibody conjugated to Alexa Fluor 488 (Invitrogen). Nuclear DAPI and viral NP staining patterns are shown in blue and green, respectively. Enlarged views are shown in red borders. Scale bar, 100 µm.
(TIF)

Figure S2 **The expression of GFP derived from NA-deficient influenza virus overlapped with the localization of NP.** MDCK cells were infected with NA-deficient influenza viruses at MOI of 0.0001. After incubation at 37°C for 48 hours, immunofluorescence analyses were performed using anti-NP antibody. Scale bar, 100 µm.
(TIF)

Figure S3 **Influenza virus A/Udorn/72 was sensitive to oseltamivir.** MDCK cells were infected with influenza virus A/Udorn/72 at a MOI of 0.001 PFU per cell. At 36 hpi, the culture supernatant was collected, and then its virus titer was determined by plaque assays. Each result was represented by a value relative to that in the absence of the drug. Error bars indicate s.d. from 3 independent experiments.
(TIF)

Video S1 **NA-deficient influenza virus spreads through cell-to-cell transmission.** Confluent MDCK cells were infected with NA-deficient influenza virus at MOI of 0.0001 in the presence of trypsin. After incubation at 37°C for 24 hours, a single GFP-positive cell and its vicinity were traced it during the period from 24 hpi to 48 hpi at interval of 1 hour. Live cell imaging data analyses was performed by Biostation ID (GE healthcare). Scale bar, 50 µm.
(MOV)

Video S2 **NA-deficient influenza virus does not spread in the absence of trypsin.** Confluent MDCK cells were infected with the NA-deficient influenza virus at MOI of 0.0001 in the absence of trypsin. After incubation at 37°C for 24 hours, a single GFP-positive cell was detected, and then this cell and neighborhood cells was traced during the period from 24 hpi to 48 hpi at interval of 1 hour. Live cell imaging data analyses were performed by Biostation ID (GE healthcare). Scale bar, 50 µm.
(MOV)
