# Supplementary figures and images for: Tamiflu-Resistant but HA-Mediated Cell-to-Cell Transmission through Apical Membranes of Cell-Associated Influenza Viruses
Source: PLoS One. 2011 Nov 30;6(11):e28178. doi: 10.1371/journal.pone.0028178 (PMC3227662; doi:10.1371/journal.pone.0028178)

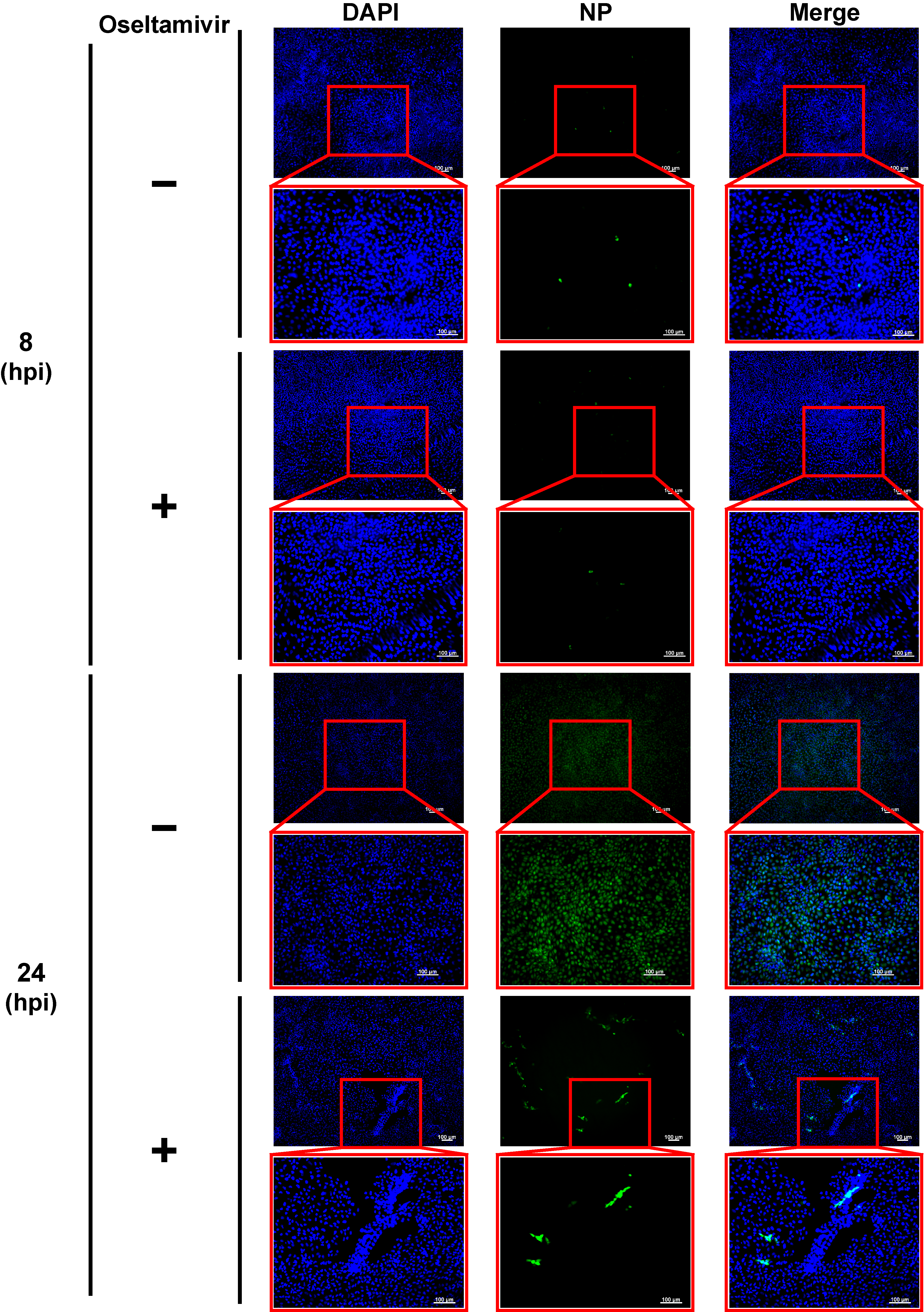

Supplement: Figure S1 — Formation of cell cluster caused by initial infection. MDCK cells were infected with influenza virus A/WSN/33 at moi of 0.0003 in the presence or absence of 50 µg/ml oseltamivir phosphate. After incubation for 8 and 24 h, immunofluorescence analyses were performed using anti-NP antibody and anti-rabbit IgG antibody conjugated to Alexa Fluor 488 (Invitrogen). Nuclear DAPI and viral NP staining patterns are shown in blue and green, respectively. Enlarged views are shown in red borders. Scale bar, 100 µm. (TIF) [file pone.0028178.s001.tif]

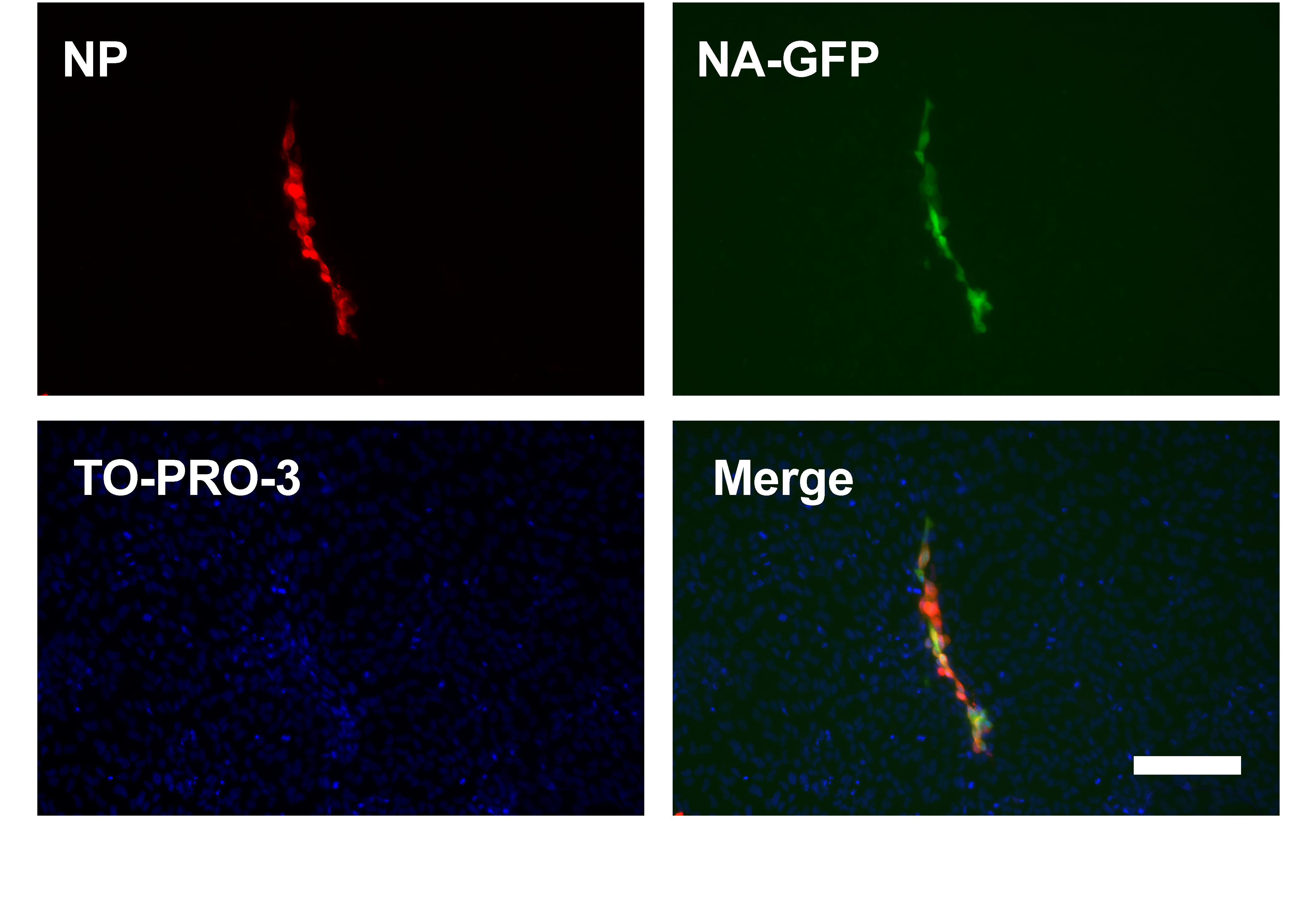

Supplement: Figure S2 — The expression of GFP derived from NA-deficient influenza virus overlapped with the localization of NP. MDCK cells were infected with NA-deficient influenza viruses at MOI of 0.0001. After incubation at 37°C for 48 hours, immunofluorescence analyses were performed using anti-NP antibody. Scale bar, 100 µm. (TIF) [file pone.0028178.s002.tif]

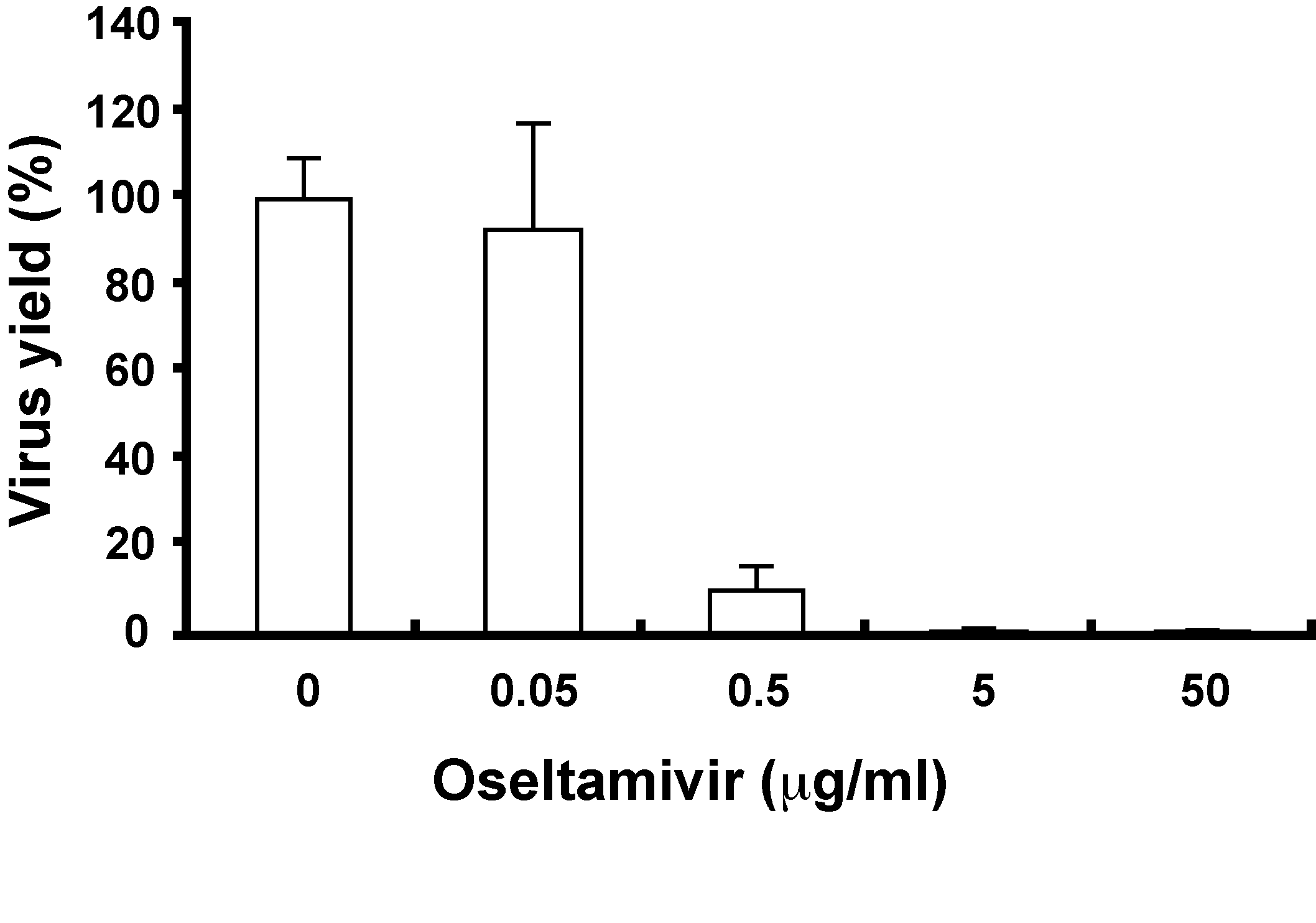

Supplement: Figure S3 — Influenza virus A/Udorn/72 was sensitive to oseltamivir. MDCK cells were infected with influenza virus A/Udorn/72 at a MOI of 0.001 PFU per cell. At 36 hpi, the culture supernatant was collected, and then its virus titer was determined by plaque assays. Each result was represented by a value relative to that in the absence of the drug. Error bars indicate s.d. from 3 independent experiments. (TIF) [file pone.0028178.s003.tif]
